# Papillary Thyroid Carcinoma in Struma Ovarii

**DOI:** 10.7759/cureus.7582

**Published:** 2020-04-07

**Authors:** Pooja Devi, Maryam Aghighi, Nagy Mikhail

**Affiliations:** 1 Pathology, Saint Barnabas Medical Center, Robert Wood Johnson Barnabas Health, Livingston, USA; 2 Pathology, Rutgers Robert Wood Johnson Medical School, New Brunswick, USA

**Keywords:** teratoma, germ cell tumor, malignant struma ovarii

## Abstract

Struma ovarii is a variant of a germ cell tumor composed predominantly of thyroid tissue. It is most often unilateral. The incidence of malignancy arising in patients with struma ovarii is rare. Here, we present a case of struma ovarii in a female presented with abdominal distension. The patient was treated with a total hysterectomy and bilateral salpingo-oophorectomy, which revealed an enlarged cystically dilated ovary. Histopathologic examination showed mature thyroid follicles with abundant colloid consistent with struma ovarii and focal area with nuclear features of papillary thyroid carcinoma. No other teratomatous elements were identified. Thyroid hormone levels were within their respective reference ranges. A diagnosis of struma ovarii should be considered in the differential diagnosis of pelvic masses in peri- and postmenopausal patients.

## Introduction

Germ cell tumors originate from the primordial germ cells, which are around 20%-25% of all benign and malignant ovarian neoplasms of germ cell origin [1]. Mature teratomas are the most common germ cell tumors. Teratomas are composed of a variety of elements such as skin, bone, hairs, and thyroid tissue. Struma ovarii, first defined by Bottlin in 1888 and, later, by Pick in 1902, is a rare monodermal variant of ovarian teratoma [2-3]. It represents approximately 2% of all mature teratomas [4].

## Case presentation

A 50-year-old, nulliparous female presented at the gynecology clinic with the complaint of abdominal distension and weight gain from the last few months. She never had a pap smear or mammogram. Her past medical history was unremarkable. There was no known history of gynecological malignancies in the family. She was vitally stable. Abdominal examination revealed a palpable abdominal mass and distension. Routine blood workup showed normal complete blood count and cancer antigen 125 (CA 125) 10 U/ml. Abdominal ultrasound showed a large solid cystic mass in the left adnexal region. The patient underwent total abdominal hysterectomy (TAH) and bilateral salpingo-oophorectomy.

**Figure 1:**
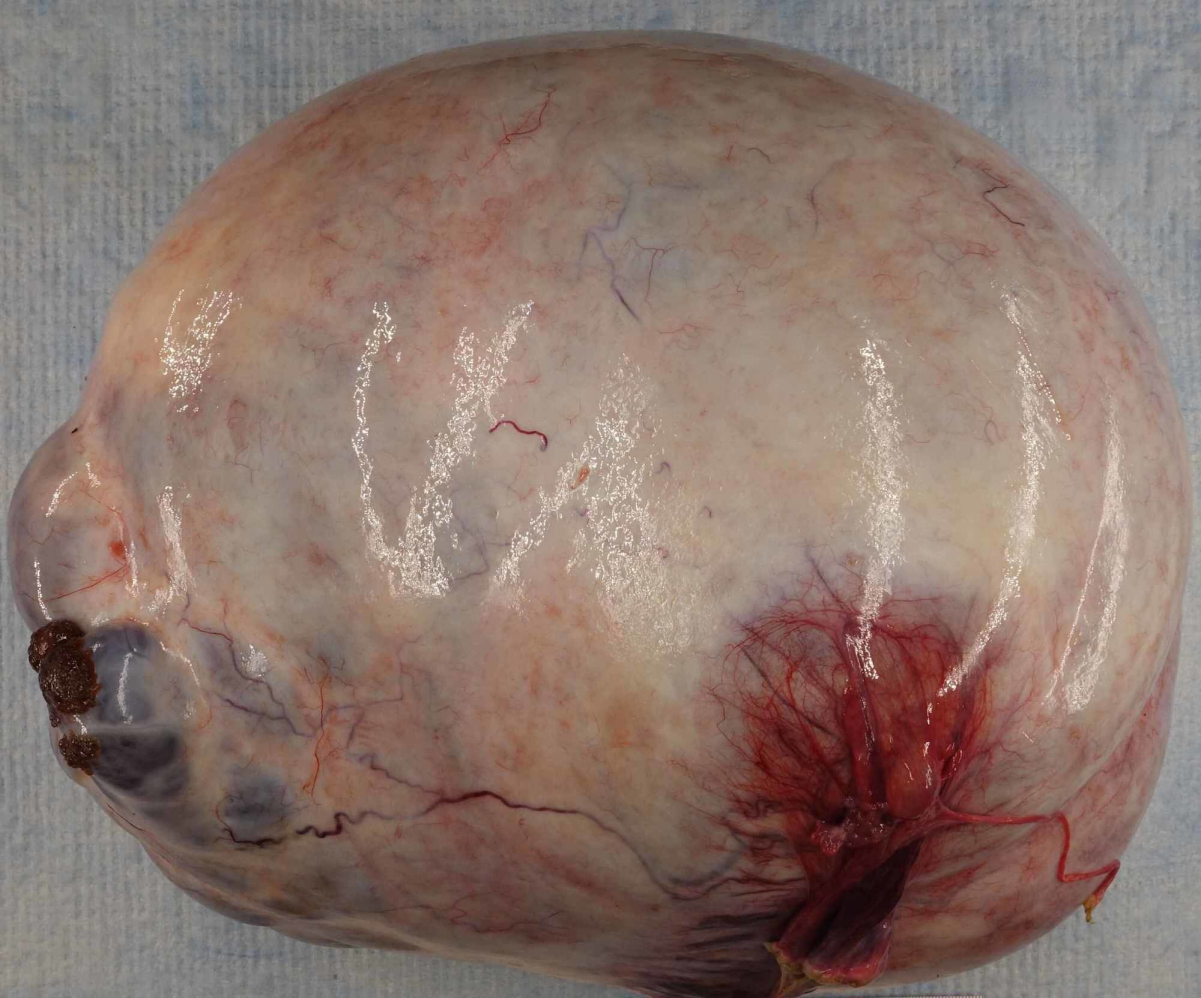
Macroscopic examination of the left ovary showed a thin-walled, multilocular cyst, 35 cm in the largest dimension and filled with clear, amber-colored fluid

Gross examination of the left ovary revealed a multiloculated complex cystic ovary with solid areas. The ovary measured 35 cm and weighed 9.6 kg (Figure 1). The capsule was smooth and intact; the cysts were filled with amber color fluid. The fallopian tubes, right ovary, and uterus were unremarkable.

**Figure 2:**
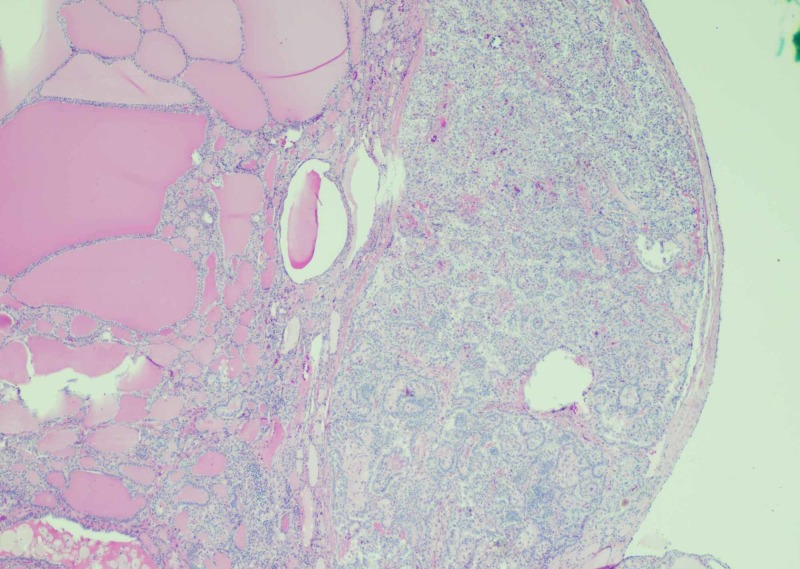
Struma ovarii with papillary thyroid carcinoma (H&E 4X) The low-power view illustrates thyroid follicles filled with colloid and a single 3.5 mm focus with increased cellularity, crowding, and nuclear alteration.

**Figure 3:**
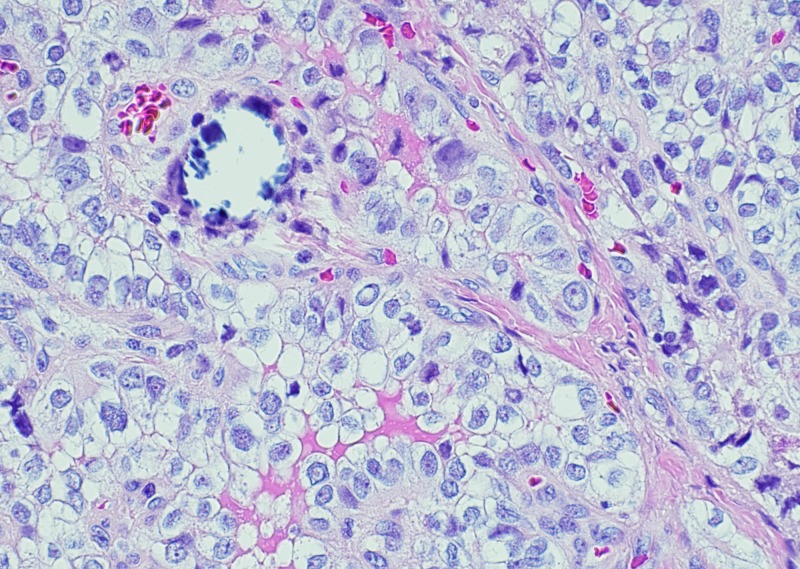
Papillary thyroid carcinoma in struma ovarii (H&E 20X) The high-power view illustrates nuclear inclusions, nuclear grooves, and focal calcification.

**Figure 4:**
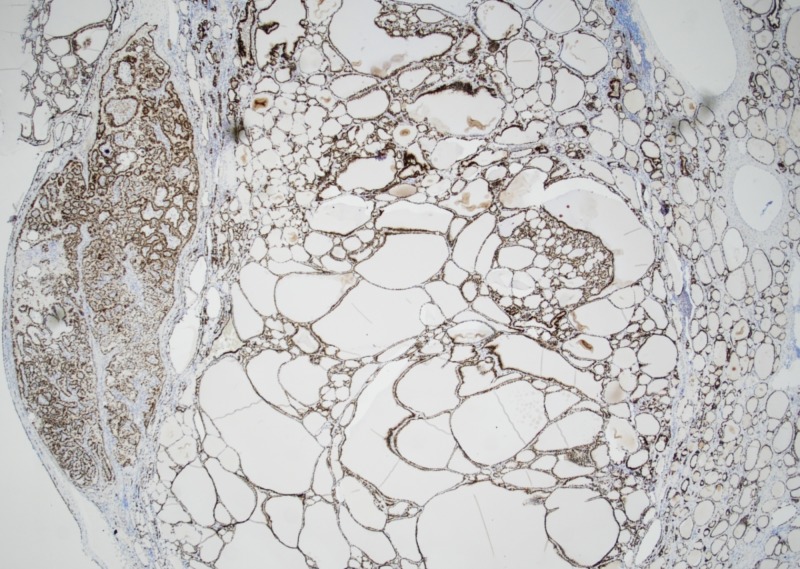
TTF-1 immunohistochemical stain shows nuclear staining of thyroid follicular cells TTF-1: thyroid transcription factor

**Figure 5:**
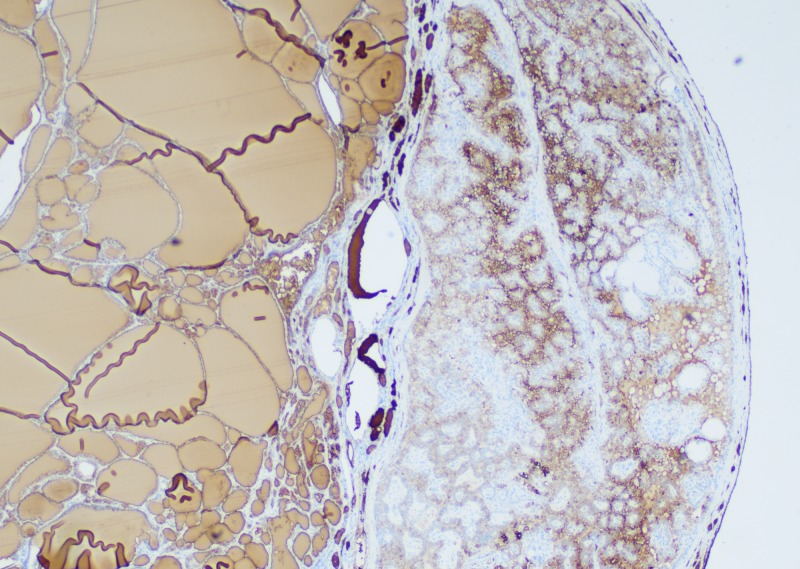
Immunohistochemical stain for thyroglobulin highlighting the thyroid component of struma ovarii

Histopathological examination revealed benign thyroid follicles with abundant colloid with a single 3.5 mm focus with nuclear inclusion, grooves, and papillary architecture in a background of serous cystadenoma (Figures 2-3). Thyroid transcription factor (TTF-1) immunostain showed the nuclear staining of thyroid follicular cells and immunohistochemical stain for thyroglobulin highlighted thyroid component of struma ovarii (Figures 4-5). No other teratomatous tissue were identified. A diagnosis of monodermal mature teratoma (struma ovarii) with papillary microcarcinoma was made. Postoperatively, the patient remained stable with the levels of normal thyroid hormones.

## Discussion

A teratoma is composed of different components. It is categorized as struma ovarii when it has at least 50% mature thyroid tissue. Although strums ovarii is usually benign, there might be a small chance of malignancy [5]. We report a case of incidental papillary thyroid microcarcinoma arising in the struma ovarii in the background of serous cystadenoma. While a papillary thyroid carcinoma is the dominant type of malignancy, follicular and other types of carcinomas may happen as well.

Struma ovarii is usually diagnosed incidentally. It is commonly asymptomatic and may be found by routine ultrasound. Other clinical findings could be abdominal mass, abdominal pain, ascites, and vaginal bleeding. It is uncommon for a patient with struma ovarii to present with hyperthyroidism features. There is a small chance of the presence of a tumor in the peritoneum [6].

The hematoxylin and eosin (H&E) stain is the best way to diagnose papillary thyroid carcinoma. The diagnosis is based on the presence of ground-glass nuclei, nuclear pseudo inclusions, and grooves. TTF-1 and thyroglobulin immunohistochemical stains can help in confirming the diagnosis [7].

In malignant struma ovarii, *BRAF*, *HRAS*, and *NRAS* point mutations and *RET* rearrangements are among several molecular abnormalities. However, the importance of these molecular abnormalities on the prognosis of this tumor is unknown [8].

It has been shown that papillary thyroid carcinoma in struma ovarii has a good prognosis with a high survival rate. Complete resection of the tumor is the treatment of choice. Other procedures like total abdominal hysterectomy and bilateral oophorectomy should be considered as well. There are different recommendations for postoperative patient management, including thyroidectomy and iodine therapy for all the cases versus recurrence, metastasis, or residual disease after operation [9].

## Conclusions

Malignant struma ovarii is a less common gynecologic cancer with no strict criteria for diagnosis. The diagnosis must be made by histopathological examination, as diagnosis preoperatively and intraoperatively is not reliable. More data are needed to determine the diagnosis, management, and follow-up for these patients.
